# Demonstrating public health impacts of translational science at the clinical and translational science collaborative (CTSC) of northern Ohio: a mixed-methods approach using the translational science benefits model

**DOI:** 10.3389/fpubh.2025.1560751

**Published:** 2025-08-13

**Authors:** Lixin Zhang, Umut A. Gurkan, Kelli Qua, Shannon Swiatkowski, Sheree Hemphill, Clara M. Pelfrey

**Affiliations:** ^1^Clinical and Translational Science Collaborative of Northern Ohio, Case Western Reserve University School of Medicine, Cleveland, OH, United States; ^2^Department of Mechanical and Aerospace Engineering, Case School of Engineering, Case Western Reserve University, Cleveland, OH, United States; ^3^Center for Medical Education, Case Western Reserve University School of Medicine, Cleveland, OH, United States

**Keywords:** translational science, translational science benefits model (TSBM), public health, policy, impact evaluation, translational science case studies, mixed method research

## Abstract

The Clinical and Translational Science Award (CTSA) program, funded by the National Center for Advancing Translational Sciences (NCATS), aims to accelerate the translation of research into public health impacts. However, measuring the societal impact of translational research poses challenges due to extended timelines for implementation. This study uses the Translational Science Benefits Model (TSBM) to evaluate the societal impact of CTSA-supported research at the Clinical and Translational Science Collaborative (CTSC) of Northern Ohio at Case Western Reserve University (CWRU). Using the TSBM, we asked how investigators have used the CTSC to demonstrate translational science impacts in public health practice both domestically and internationally. Using a mixed methods approach, this study analyzed TSBM-based survey data from CTSC-supported KL2 Scholars and Pilot Program awardees, along with key publications and interviews, to document societal benefits across four TSBM domains: Clinical & Medical, Community & Public Health, Economic, and Policy & Legislative. Findings demonstrate that CTSC-supported research improved public health by enhancing healthcare access, improving health outcomes, informing policy, and generating economic benefits. These impacts span local, national, and global contexts. By applying a mixed methods approach, we demonstrate the value of using the TSBM not just as an evaluative framework, but as a strategic tool for capturing the real-world significance of translational science. This approach strengthens the ability of CTSA hubs to highlight the broader public value of their work, reinforcing the CTSA program’s mission to transform scientific discoveries into lasting health and societal benefits.

## 1 Introduction

The Clinical and Translational Science Award (CTSA) program, funded by the National Center for Advancing Translational Sciences (NCATS), aims to accelerate turning scientific discoveries into actionable solutions that improve human health. The CTSA is committed to improving health outcomes, increasing access to quality healthcare, and fostering advancements that benefit all populations. However, effectively demonstrating these societal impacts remains challenging. Translational research often requires long-term implementation and validation to produce measurable health benefits, making it difficult to immediately quantify the public health gains from CTSA-supported research. This difficulty is particularly significant in assessing health impact, where translating findings into improved health outcomes require sustained efforts that are difficult to measure over short periods.

To address the need for more robust evaluation of translational science’s real-world impacts, the Translational Science Benefits Model (TSBM) was developed as a structured framework to document the societal benefits derived from translational research efforts. TSBM identifies and categorizes translational impacts across four domains: Clinical & Medical, Community & Public Health, Economic, and Policy & Legislative (1). The TSBM enables researchers to capture specific, tangible examples of societal benefits, enhancing transparency and accountability in reporting the impacts of research. This approach is valuable for evaluating CTSA programs, where documenting health improvements across these domains can yield comprehensive insights. Despite its potential, empirical evidence on the TSBM’s applicability within real-world CTSA environments remains limited, especially regarding its effectiveness in public health impacts.

The CTSC of Northern Ohio plays a vital role in advancing translational research that addresses health challenges that affect people of all backgrounds. Through its KL2 Scholars and Pilot Grant programs, the CTSC supports early-career researchers and innovative projects aimed at improving health and healthcare access. The KL2 program provides tailored training and mentorship, while the Pilot Program funds high-impact studies targeting critical health issues in all communities. Together, these programs foster collaboration and drive health outcomes through translational science.

However, capturing the real-world impact of such research requires more than simply categorizing outputs. Traditional methods, such as surveys or administrative metrics, often lack the nuance needed to fully explain how research leads to meaningful societal change. Surveys alone can produce limited or outdated insights and often miss the context-specific pathways through which translational work achieves impact. Moreover, quantitative data may obscure critical mechanisms—such as stakeholder engagement or community partnerships—that are essential to translational success but difficult to measure with standardized tools.

To overcome these limitations, this paper first leverages the TSBM to assess the societal impact of translational research supported by the CTSC. We focused on projects led by CTSC-supported KL2 Scholars and Pilot Program awardees, investigating how these initiatives have advanced public health outcomes both locally and globally. We employed a mixed-methods approach, supplementing TSBM with semi-structured interviews to capture deeper insights into how these projects addressed public health challenges. This approach allows us to demonstrate the TSBM’s utility while highlighting the public health impacts of research supported by the CTSC.

## 2 Materials and methods

**Figure 1:**
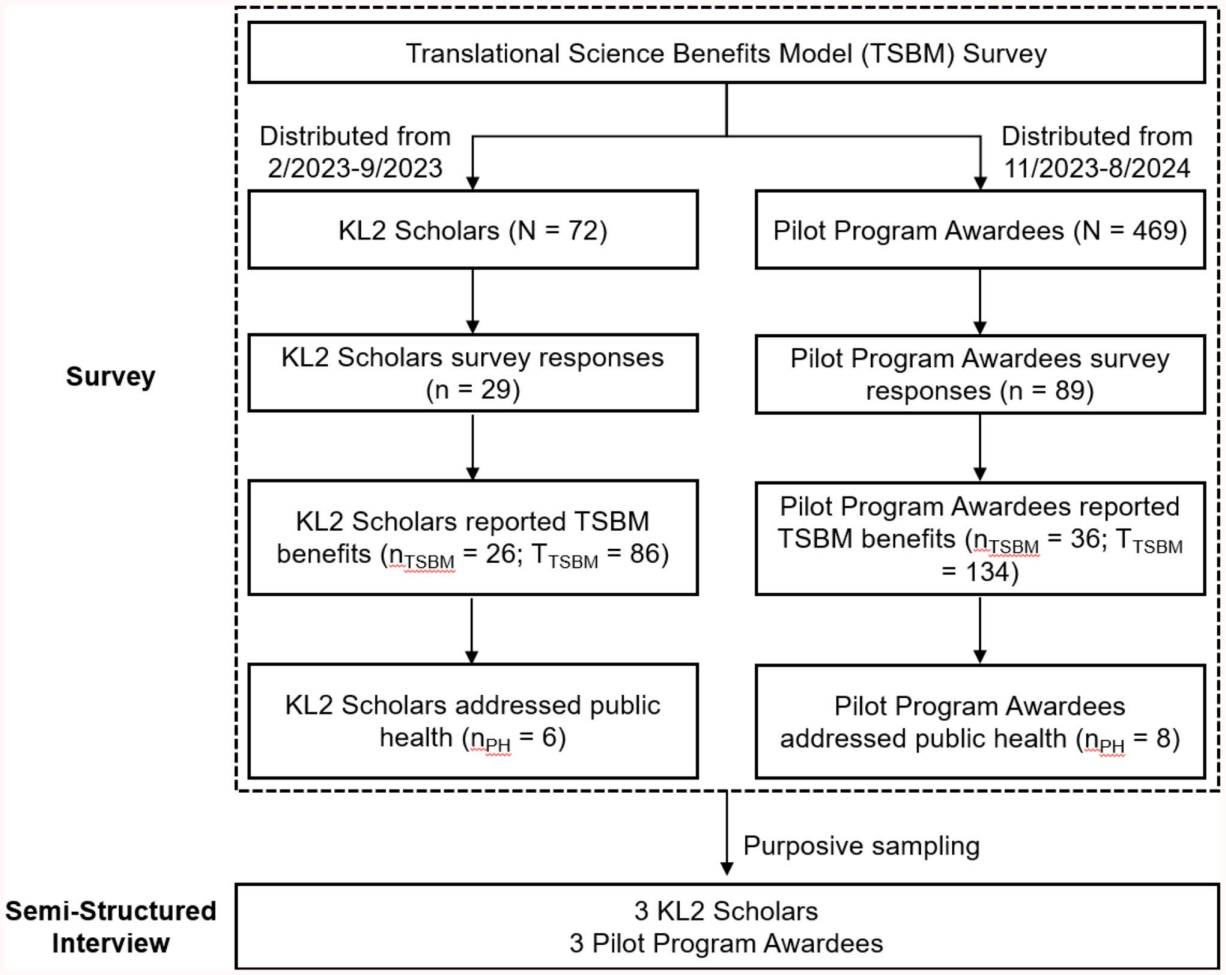
Flowchart of translational science benefits model (TSBM) survey and semi-structured interviews. N: Total number of participants invited to complete the survey. n: Total number of survey respondents. n_TSBM:_ Number of survey respondents who reported at least one TSBM benefit. T_TSBM_: Total number of TSBM benefits reported by the group. n_PH:_ Number of survey respondents addressing public health.

This study utilized a mixed-methods approach to evaluate the translational benefits of research supported by the CTSC. Figure 1 presents a flowchart outlining the process of the TSBM survey and semi-structured interviews with selected KL2 Scholars and Pilot Program awardees. By integrating survey data with interview results, the study provides a multidimensional evaluation of how CTSC-supported research drives advancements in public health.

### 2.1 Study participants

Participants were selected through a multi-step process designed to identify researchers whose work embodies the translational goals of the CTSC. This process, detailed below, ensured the broad participation of researchers with demonstrable contributions to advancing public health.

The first phase involved administering surveys to two groups: former KL2 Scholars (*n* = 72) and Pilot Program awardees (*n* = 469) from 2008 to 2022. These groups were selected for their varied research projects and potential for translational impact. By including these distinct groups, the analysis captured a broad spectrum of research areas, reflecting the CTSC’s commitment to bridging scientific discovery and societal benefit.

To complement the survey data, a purposive sampling strategy was employed to identify survey responses that showed significant contributions to public health advancements. The selection process adhered to stringent criteria, ensuring the reliability, relevance, and impact of the chosen examples:

- **Substantive Detail and Documentation**: Respondents who selected “Demonstrated” for a TSBM indicator were required to provide supporting evidence (e.g., publications, URLs, or CVs). Only responses with sufficient detail and credible documentation were included. Two research team members independently verified outcomes to ensure reliability.
- **Varied Beneficiaries**: Selected examples highlighted benefits for populations experiencing health challenges unique to their situation (e.g., pregnant women, intimate partner violence survivors, Medicaid recipients), international populations (e.g., those in low resource countries with genetic disorders or parasitic infections), rural populations, and professions at high-risk of developing cancer.
- **Contextual Breadth**: Selected research represented societal benefits achieved at local, national, and international levels, ensuring a comprehensive view of translational impact.
- **Scalability**: Projects demonstrated the potential for replication and implementation across numerous healthcare settings, considering economic and logistical feasibility.
- **Multidisciplinary Engagement**: The research demonstrated collaboration among healthcare providers, local organizations, policymakers, and academic researchers, highlighting the importance of interdisciplinary approaches.

This approach identified six high-impact researchers based locally in Cleveland whose work exemplified translational success in addressing critical health challenges. These researchers were invited to participate in semi-structured interviews to explore the pathways through which their research advanced public health outcomes.

### 2.2 Survey instrument

The survey instrument for this study was developed using the TSBM, a framework for evaluating the societal benefits of translational research (1). This model systematically captures many different outcomes, including policy influence, public health advancements, and other health outcomes. It served as the foundation for constructing a robust quantitative dataset to evaluate the impact of CTSC-supported research.

The survey was designed using the REDCap Electronic Data Capture platform (2) and incorporated the TSBM indicators (1). In the survey after each indicator, respondents were asked whether the indicated benefit was “Demonstrated” or “Potential.” If “Demonstrated” was selected, branching logic opened a text box where respondents were required to “Briefly describe demonstrated [benefits] using lay language. Please provide evidence or URL link, if available (e.g., published materials).” Examples of documented evidence may include, but are not limited to, published policy documents, press releases, newspaper articles, white papers, collaborating or corroborating research studies that have been published, regulatory approval, governmental bills and laws, formal records documenting grants or milestones, or review articles (e.g., meta-analyses and scientific reviews). The survey was administered to two distinct groups: KL2 Scholar trainees and Pilot Program awardees. For the KL2 Scholar trainees, the survey was conducted between February and September 2023, while the survey for Pilot Program awardees took place between November 2023 and August 2024. The extended administration period for the Pilot Program was necessary due to the nature of the program, with several investigators having received up to five different pilot grants between 2008 to 2022. To avoid survey fatigue, surveys were distributed in non-overlapping periods, preventing any single investigator from receiving multiple surveys at the same time. Both survey instruments can be found in Appendix A.

### 2.3 Semi-structured interview protocol

To complement the quantitative analysis, semi-structured interviews were conducted with researchers whose work demonstrated measurable contributions to public health. All participants were consented under CWRU IRB STUDY20241228 to use their names, survey responses and interview material in constructing case examples. The interviews aimed to uncover the processes and mechanisms through which their research influenced societal outcomes, with a focus on public health impacts and the CTSC’s role in supporting these impacts. This qualitative approach emphasized actionable insights and a deeper understanding of researchers’ experiences. The interview protocol is provided in Appendix B. Additionally, research databases such as Overton (3) and Dimensions (4) were used to supplement the qualitative data by tracking the influence of CTSC-supported research on policy and legislation, highlighting the broader implications of translational efforts for public health. Overton Index is the world’s largest policy and gray literature database (3). Dimensions. AI, part of Digital Science, is a comprehensive database and platform focused on research and innovation. Dimensions was used to link research data, including publications, patents, media coverage and policy documents (4).

## 3 Results

### 3.1 Survey results

Survey responses were collected, analyzed, and categorized into the four major TSBM domains: Clinical & Medical, Community & Public Health, Economic, and Policy & Legislative. KL2 Scholars had a response rate of 29 out of 72 (40%). Among the respondents, 26 out of 29 (90%) reported a total of 86 demonstrated benefits across the four TSBM domains. Pilot Program Awardees had a response rate of 89 out of 469 (18%). Of the 89 respondents, 36 (40%) reported a total of 134 demonstrated benefits across the four TSBM domains.

**Figure 2:**
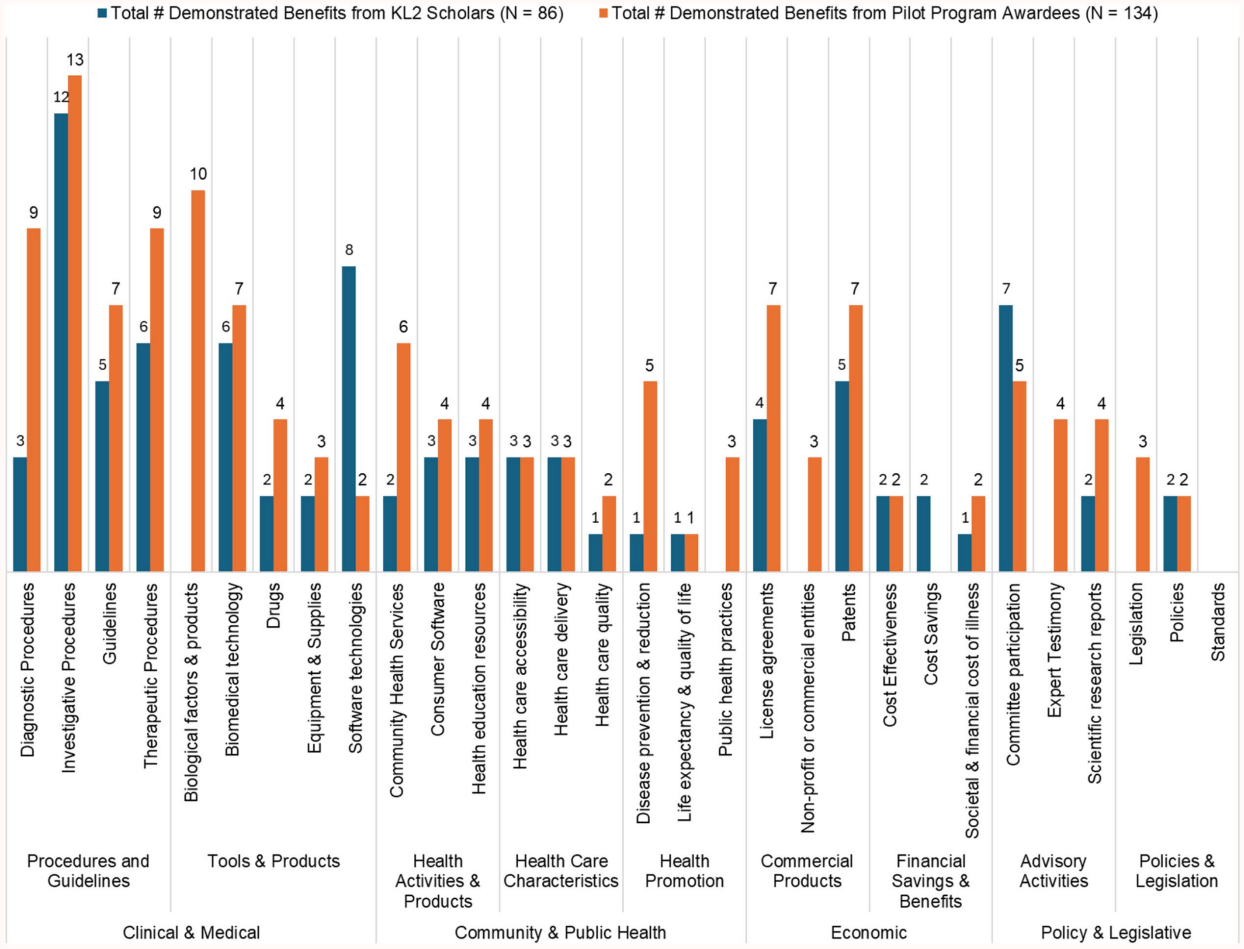
Demonstrated TSBM benefits from CTSC-supported 26 KL2 scholars and 36 pilot program awardees.

Figure 2 highlights the translational outcomes achieved by CTSC-supported KL2 Scholars and Pilot Program Awardees, with demonstrated benefits across clinical, public health, economic, and policy domains.

- **Clinical & Medical Contributions**: CTSC-supported research has led to 64 demonstrated benefits in diagnostic, therapeutic, and investigative procedures, directly enhancing clinical practices and patient care. Additionally, researchers developed 10 biological products, 13 biomedical technologies, and 10 software tools.
- **Community & Public Health**: These researchers have made meaningful strides in public health, with 8 contributions to local health services, 7 health education resources, and 15 improvements in healthcare access, delivery, and quality. Research efforts addressing disease prevention, quality of life and public health practices resulted in 11 reported benefits.
- **Economic Benefits**: Economic outcomes from CTSC-supported research include 12 patents, 11 license agreements, and the establishment of 3 commercial or non-profit entities. Additionally, contributions to cost savings and cost-effectiveness highlight the financial value of translational research.
- **Policy and Legislative Influence**: CTSC researchers reported policy contributions, with 22 advisory activities, including committee participation and expert testimony, and 7 outcomes influencing policy and legislation.

### 3.2 TSBM case study results

The six cases highlighted below were constructed from both survey data and interviews and demonstrate the potential of translational science to address obstacles to health and improve public health outcomes. These researchers, supported by the CTSC, underscore the critical role of dedicated resources, interdisciplinary approaches, and community-centered solutions in achieving meaningful impact. The findings offer valuable insights into the mechanisms of success, the challenges faced, and the future directions needed to expand the reach and efficacy of translational science initiatives.

#### 3.2.1 Case study 1. *The essence of primary care*, Shari Bolen, MD, MPH

##### 3.2.1.1 Utilization of the CTSC

Bolen’s research has been significantly influenced by her extensive engagement with CTSC resources. As a KL2 scholar, Bolen benefited from protected time to deepen her expertise in mixed-methods research, essential for the nuanced exploration of healthcare interventions. The CTSC’s support facilitated her participation in meetings, which, as she described, “*brought all of the scholars together and supported a transdisciplinary model.*” These collaborative experiences laid a solid foundation for her work on complex healthcare challenges.

After completing the KL2 program, Bolen continued to leverage CTSC resources, such as the REDCap data management tool, to allow efficient management of large datasets. The CTSC informatics module played a critical role in helping Bolen design and implement innovative health information dashboards. These tools have been pivotal to her work, especially in monitoring and improving health outcomes across affected communities.

##### 3.2.1.2 Societal benefits in TSBM categories

Bolen’s research demonstrates substantial societal benefits across multiple TSBM domains:

**Clinical & Medical Benefits**: The innovative use of the positive deviance approach in primary care has revolutionized hypertension management, addressing one of the most prevalent and challenging chronic conditions (5, 6). This approach identifies high-performing clinics or communities—outliers that have achieved exceptional success in blood pressure control—analyzes their strategies and adapts these best practices to drive improvements across other primary care settings.

**Community & Public Health Benefits**: Bolen’s development of comprehensive health education resources supports ongoing education of healthcare providers and local health workers (personal communication, November 2024). Bolen’s initiative has been disseminated widely, enhancing the overall quality of healthcare delivery and facilitating widespread adoption of best practices in patient care.

**Policy & Legislative Benefits**: Bolen has influenced health policy and legislative outcomes at both state and national levels, championing universal reforms to expand healthcare access and improve outcomes for all populations. Through strategic advisory roles, innovative research, and collaboration with key stakeholders, she has addressed immediate policy challenges while laying the groundwork for long-term change in chronic disease management.

In her advisory roles, Bolen has provided strategic leadership on national committees that shape health policy. As a member of the National Clinical Care Commission (NCCC), she contributed to a landmark congressional report that identified critical gaps in federal diabetes policies and offered actionable solutions to improve care delivery for millions (7, 8). Her participation in the CDC National Hypertension Roundtable further underscores her role as a national leader, where she developed strategies to enhance hypertension prevention and treatment, directly addressing public health (personal communication, November 2024).

Bolen’s ability to translate research into policy has been pivotal in driving widespread change. Her publications in Diabetes Care have informed federal diabetes policies (7, 8), while her Medicaid-focused research (9, 10) have led to significant reforms in healthcare delivery. These efforts positioned her as a trusted resource for bridging the gap between research and regulation.

Bolen’s policy achievements are particularly notable in chronic disease management. She has driven Medicaid reforms that expanded coverage for research-informed interventions such as the Diabetes Prevention Program and Diabetes Self-Management Education, ensuring patients have access to preventive care. She also advanced efforts to increase access to diabetes technology, such as continuous glucose monitors, and improving care for countless patients. These accomplishments, supported by her research funded by the Ohio Department of Medicaid, demonstrate the tangible benefits of her policy leadership (personal communication, November 2024).

Beyond diabetes, Bolen’s work in hypertension policy has shaped state and federal initiatives to incorporate best practices into routine primary care. Her strategies have been instrumental in advancing blood pressure control across all populations, further reinforcing her impact on chronic disease prevention and management (11).

**Policy Citations**: Table 1 provides a global overview of policy documents from 17 different countries that cite Bolen’s research, highlighting the interplay between research, guidance, and practice in shaping healthcare policy. The findings reveal a significant concentration of healthcare policy contributions in publications (60%) and clinical guidance (27%), underscoring their importance in advancing global health practices and recommendations.

**Table 1:** Policy citations of Bolen’s research, by country*.

Source country	Policy document type					Grand total
	Clinical guidance	Legal documents	Publication	Scholarly article	Working paper	
Australia			4			4
Canada	1		1	1		3
Colombia			1			1
France	1					1
Germany	1					1
IGO			7		2	9
Ireland			1			1
Kosovo			1			1
Malaysia			2			2
Netherlands			1			1
Nigeria			1			1
Norway	1					1
Peru			2			2
Portugal				1		1
Spain			2	3		5
Sweden	1	1	2			4
Switzerland			1			1
UK	4		1			5
USA	7		9			16
Grand Total	16	1	36	5	2	60

*Retrieved from https://www.overton.io/ on December 1, 2024.

##### 3.2.1.3 Populations affected, geographical impact and research expansion

**Populations Affected**: Bolen’s research focuses on improving healthcare access and outcomes for Medicaid-covered individuals in Ohio, particularly those facing significant economic challenges and chronic conditions like diabetes and hypertension. Her work directly impacts healthcare outcomes by targeting roadblocks that prevent timely and effective care for all populations. By tailoring interventions for both rural and urban communities, Bolen ensures that all populations across Ohio benefit from improved healthcare access and chronic disease management support.

**Geographic Focus and Impact**: Bolen’s research primarily targets northeastern Ohio and Cuyahoga County, regions with disproportionately high rates of hypertension and cardiovascular diseases. Collaborating with local health organizations and policymakers, she identifies communities with the greatest need and implements data-driven, community-based solutions. Programs such as Cardi-OH (12) bridge the gap between healthcare providers, Medicaid enrollees, and community resources. Additionally, programs such as Better Health Partnership deliver research-informed healthcare improvements, addressing system challenges and reaching thousands of individuals across Ohio, including urban and rural communities in Cleveland (13).

**Focus on High-Risk Populations**: Bolen’s work particularly focuses on addressing the needs of high-risk populations who experience higher rates of chronic diseases like hypertension and diabetes and disproportionately worse health outcomes (14). By leveraging community engagement and data-driven approaches, Bolen ensures that healthcare strategies are relevant and responsive to the specific needs of these populations in both rural and urban areas. Her collaborations with the Northeast Ohio Medical University (NEOMED) to establish the Medicaid Technical Assistance and Policy Program funded Northeast Ohio Quality Improvement (QI) hub further amplify her impact by enhancing quality improvement efforts and community outreach (personal communication November 2024).

##### 3.2.1.4 Advancements in public health

Bolen’s research has significantly contributed to developing targeted interventions within primary care practices to improve health outcomes. A key highlight is the use of the positive deviance approach to improve blood pressure control among high-risk populations. This work focused on low-performing practices that served economically challenged populations. By implementing varying intensities of research-informed care strategies, the project significantly reduced variance in blood pressure control between different insured groups, such as Medicaid, Medicare, and commercial insurance (6). Recognized as a leading implementation study, this research garnered national attention (personal communication, November 2024).

##### 3.2.1.5 Expanding the research impact

Bolen’s research is evolving to meet the health needs of Ohio’s population. Central to this vision is the ongoing development of community engagement and workforce training initiatives, which enhance the capability of primary care settings to manage a broader spectrum of health issues. A key component of this effort is the integration of social determinants of health into everyday clinical practice—a step that promises to make healthcare more available.

To support these objectives, Bolen’s research team is actively seeking additional funding, particularly through Medicaid initiatives and grants from the Agency for Healthcare Research and Quality (AHRQ), to strengthen their infrastructure for community engagement and workforce development in behavioral health areas. These efforts aim to create a sustainable model that not only improves health outcomes but also prepares the healthcare system to respond to emerging health needs.

The next steps for Bolen’s research involve a dual focus: continuing to address the existing gaps in health outcomes through targeted interventions and broadening their scope to incorporate more comprehensive approaches to public health challenges. Key priorities include fostering stronger partnerships and aligning with state health improvement plans to ensure interventions are well-supported and strategically focused on Ohio’s most pressing health issues.

#### 3.2.2 Case study 2. *Development of a patient hand cleaning system for older adults in healthcare settings to support self-management*, Shanina C. Knighton, PhD, RN, CIC

##### 3.2.2.1 Utilization of the CTSC

Knighton leveraged CTSC resources to advance her research on patient hand hygiene solutions. As a KL2 Scholar, she utilized mentorship, funding, and infrastructure to dedicate 80% of her time to research focused on innovative tools addressing patient behavior and hygiene challenges in healthcare settings. The program provided her with invaluable mentorship from experts such as Drs. Mary Dolansky, Colin Drummond, and Curtis Donskey, enriching her understanding and approach to developing patient-centered interventions.

In 2020, Knighton secured a CTSC Annual Pilot grant alongside Colin Drummond to develop a “Patient Hand Cleaning and System.” This grant enabled feasibility studies for optimizing original technologies, supporting incremental progress in design, testing, and refinement of components—including a specialized bracket for bedrails and careful materials selection. This foundational work led to the NIH-funded Clean Hands Accessible and Manageable for Patients (CHAMPs) R01 study in 2023. The CHAMPs study focuses on infection prevention through a smart dispenser designed to support hand hygiene and reduce hand contamination among older adults (15). CTSC resources also facilitated access to clinical sites like MetroHealth and the Cleveland VA, enabling Knighton to conduct a randomized controlled trial aimed at reducing hand contamination. The incremental support from the CTSC was pivotal in advancing her innovative work from initial feasibility studies to a federally funded clinical trial.

##### 3.2.2.2 Societal benefits in TSBM categories

Knighton’s research demonstrated broad societal benefits within the following TSBM categories:

**Clinical and Medical Benefits**: Knighton’s work addressed gaps in patient hand hygiene practices, particularly among older adults, by focusing on identifying and mitigating challenges to infection prevention. Her clinical trials tested the effectiveness of her interventions in reducing pathogens such as MRSA, MSSA, and Enterococci on patient hands. Her contributions include the development of innovative biomedical technology, such as a patented smart dispenser that attaches to patient bedrails, providing verbal reminders to support hand hygiene, especially for patients with limited mobility (16). Additionally, she pioneered a tracking system to differentiate prompted versus unprompted hand-cleaning behaviors, offering critical insights for improving patient engagement strategies (17). Expanding her focus, Knighton and her collaborators published a paper calling for the development of wearable sensors for COVID-19 (18). This work is also cited in a wearable device patent for reducing exposure to pathogens (19). Her work is cited in international clinical guidelines for infection control (20), further underscoring her impact on global infection control practices.

**Figure 3:**
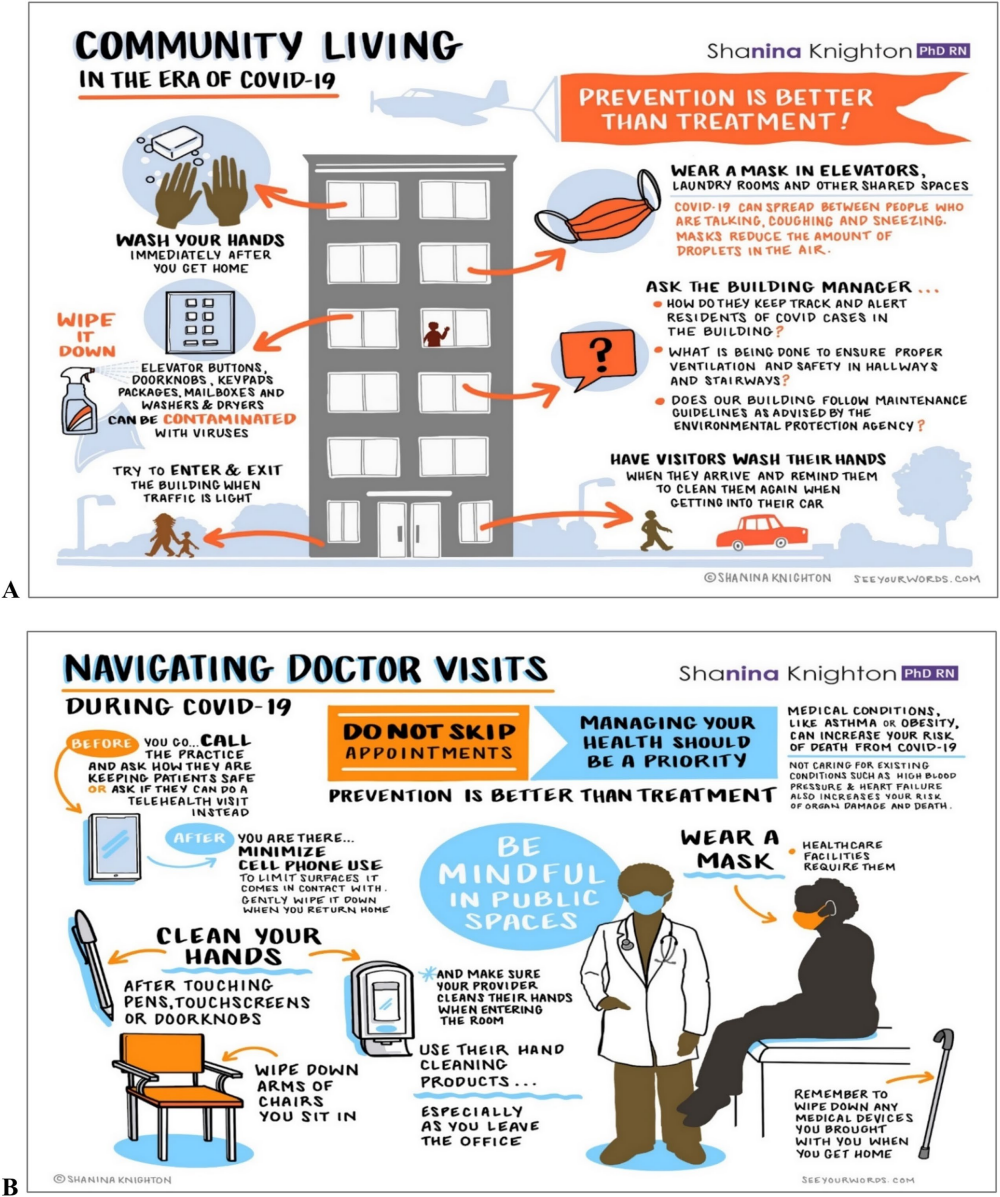
Community living in the era of COVID-19 **(A)** and navigating doctor visits in the era of COVID-19 **(B)**.

**Community and Public Health**: During the COVID-19 pandemic, Knighton expanded her research focus to address hygiene poverty in lower-income, multigenerational households. She created and disseminated over 300,000 copies of 12 sets of COVID-19 infographics nationwide, promoting actionable infection prevention education (personal communication, December 2024). These infographics offered plain language practical strategies to reduce germ exposure and transmission, particularly in public settings and multigenerational households, where hygiene imbalances are more prevalent (Figure 3). Alongside her educational efforts, her interventions, such as the smart dispenser and other educational resources, effectively reduced pathogen contamination in both healthcare and community environments. These efforts not only mitigated infection risks but also equipped communities with tools to strengthen public health practices.

**Policy and Legislative**: Knighton contributed to the drafting of Ohio’s House Bill 628 (21), calling for mandatory patient hand hygiene education in healthcare settings. The data presented in Table 2 highlights the distribution of policy document types across three countries: Germany, Ireland, and the United States.

**Table 2:** Policy citations of Knighton’s research, by country*.

Source country	Policy document type					Grand total
	Clinical guidance	Legal documents	Publication	Scholarly article	Working paper	
Germany	1					1
Ireland			1			1
USA			1			1
Grand Total	1		2			3

*Retrieved from https://www.overton.io/ on December 1, 2024.

##### 3.2.2.3 Populations affected, geographical impact and research expansion

**Populations Affected**: Knighton’s research primarily targeted older adults in acute and long-term care settings, a group at increased risk for infections due to widespread challenges and reduced access to hygiene resources. During the COVID-19 pandemic, her efforts expanded to address lower-income, multigenerational households who faced additional challenges such as hygiene poverty, economic pressures, limited access to infection prevention tools, and inadequate health education. Many participants reported challenges such as the inability to afford basic cleaning supplies or lack of time to maintain hygiene due to economic pressures (22). These populations included communities living in Cleveland’s low-resource areas, such as Buckeye-Shaker, Woodland, and Kinsman, where worse health outcomes are often found.

**Geographic Impact**: The geographic impact of Knighton’s work extends beyond Cleveland. Her educational materials and COVID-19 infographics, created in collaboration with SeeYourWords.com, were disseminated nationwide, reaching urban and rural populations, including those in Florida and Pennsylvania. Partnerships with national nurses’ organizations extended her reach to rural communities with limited hygiene resources, addressing critical gaps in infection prevention education (personal communication, December 2024).

**Research Expansion to Additional Populations**: Knighton is broadening her focus to include individuals with mobility limitations, pediatric populations, and outpatient care settings. Her work includes refining smart dispenser technology and developing tailored educational materials to meet the specific needs of these groups. Knighton continues to prioritize rural and low-income communities by leveraging public health partnerships to scale her interventions, reflecting her commitment to sustainable, long-term improvements in infection prevention.

##### 3.2.2.4 Advancements in public health

Knighton’s research tackles the root causes of infection prevention by addressing how opportunity, motivation, and capability influence hygiene behaviors at the household level. As she stated,

*“Hygiene poverty significantly impacts [high-risk] populations, and my research integrates this understanding into both technology and education to drive [system-wide] change. By addressing these factors, I aim to empower families and communities to adopt sustainable infection prevention practices….”*

Her work has made substantial contributions in improving public health outcomes by overcoming obstacles to infection prevention. Knighton developed understandable educational materials that empower populations to adopt sustainable hygiene practices. These efforts have improved access to critical hygiene tools, enabling communities to engage effectively in infection prevention measures.

A key element of Knighton’s work is her smart dispenser technology, designed to address mobility challenges and support hand hygiene. This practical solution has been effective among older adults in clinical and home settings. Highlighting its focus on usability and behavior change, Knighton observed:

“*Patients are able and willing to practice hand hygiene if they are reminded and if their hand hygiene products are conspicuously placed and easy to use. I learned from one of my studies that most patients perceive health care worker hand hygiene to be more important than their own, and that the hand hygiene products in the hospital are intended for health care workers, not for patients*” (23).

During the COVID-19 pandemic, Knighton’s commitment to improved health outcomes became even more apparent. She tackled dual challenges of health literacy and misinformation, noting:

“*In addition to the challenge of health literacy, people, during COVID-19, [we]re challenged with both misinformation and a lack of [available] visual information regarding practical infection prevention steps they can take to manage their care and quality of life…. I felt compelled to do something for communities. I wanted to walk with people in their day-to-day lives so that I could ease their stress levels by providing them with practical tools*” (23).

In response, Knighton led the creation and dissemination of carefully designed infographics targeting multigenerational households and low-resource communities. This large-scale educational effort provided actionable infection prevention strategies, bridging critical knowledge gaps and promoting behavior change.

Knighton’s work extends beyond research and education to policy reform. Her support for universal solutions, such as Ohio House Bill 628 (21), underscores her commitment to ensuring everyone has access to hygiene education and resources. By translating research into actionable policy, she has worked to improve access to hygiene materials, further advancing public health outcomes.

By focusing on high-risk populations and tailoring her interventions to their unique needs, Knighton’s work has led to measurable improvements in public health at both individual and community levels. Her approach—combining technology innovation, education, and policy promotion—serves as a model for how to improve health outcomes for all people.

##### 3.2.2.5 Expanding the research impact

Knighton’s next steps focus on scaling her smart dispenser technology across healthcare settings, community health centers, and lower-income households while developing community centered educational materials to address hygiene differences. She plans to collaborate with public health organizations to drive widespread changes targeting hygiene poverty and will expand her research to include individuals with mobility limitations, children, and rural populations. By addressing the root causes of hygiene imbalances and tailoring solutions to many different needs, Knighton aims to create sustainable improvements in infection prevention, improve health outcomes, and advance public health on a broader scale.

#### 3.2.3 Case study 3. *Do antenatal maternal infections [affect] childhood vaccination?* Indu Malhotra, PhD

##### 3.2.3.1 Utilization of the CTSC

Malhotra secured three pivotal pilot grants through the CTSC, each addressing critical aspects of maternal and child health in low-resource environments. The 2010 Core Utilization Pilot (CUP), titled “*Do antenatal maternal infections [affect] childhood vaccination?*,” investigated how maternal infections during pregnancy influence infant immune responses to vaccines. This project used biostatistical and epidemiological tools to assess complex immunological interactions. The 2012 CUP focused on the “*Effect of maternal infections on B-cell responses to vaccines mediated by infant T-cell responses to malarial antigens*.” This project employed Luminex technology to develop high-throughput diagnostics for antibodies against multiple antigens, providing a cost-effective approach to evaluating vaccine responses. In 2017, the focus shifted to the “Effect of antenatal maternal infections and anemia on childhood anemia,” examining how maternal anemia and parasitic infections impact infant health through specimen-sparing diagnostic assays.

CTSC resources enabled the development and implementation of innovative diagnostic techniques, such as the bead assay, which required minimal blood samples. This advancement was crucial in reducing the ethical and logistical challenges associated with studying nutritionally deficient and high-risk groups. Moreover, the CTSC facilitated collaborations between Malhotra’s team and Kenyan health authorities, leading to the establishment of diagnostic laboratories and training programs for local researchers and health workers. These collaborations were instrumental in enhancing healthcare infrastructure in the region, ensuring the sustainability of the project’s impact (24, 25).

##### 3.2.3.2 Societal benefits in TSBM categories

Malhotra’s research yielded multifaceted societal benefits:

**Clinical & Medical Benefits**: The research introduced novel diagnostic and therapeutic interventions tailored to resource-limited settings. The team developed a multiplex flow immunoassay capable of detecting antibodies against parasitic infections such as lymphatic filariasis and schistosomiasis using minimal blood samples. This high-throughput assay significantly improved diagnostic accuracy while minimizing the invasiveness of testing procedures. Similarly, Luminex assays were developed to test for the presence of antibodies to malaria and childhood vaccines. Therapeutic protocols were equally innovative, incorporating prophylaxis for malaria, intestinal helminths, and other parasitic diseases. These interventions markedly improved maternal health, reducing risks of adverse pregnancy outcomes and creating a healthier start for newborns (25, 26).

**Community & Public Health Benefits**: The research also delivered significant public health benefits. Conducted in rural areas of Kwale District, the study addressed the needs of populations with limited access to healthcare. Community health workers were trained to educate mothers on the importance of antenatal care, proper sanitation, and regular vaccinations for their children. This grassroots outreach model effectively increased compliance with vaccination schedules, which now exceeds 90% among the study population, reducing the prevalence of malaria. Mothers who could not visit healthcare facilities were reached through home visits, ensuring comprehensive community participation (24, 27).

**Policy & Legislative Benefits**: The research produced actionable insights that informed national health policies in Kenya. Findings highlighted the economic and societal costs of untreated maternal parasitic infections, leading to the integration of preventive therapies into routine healthcare guidelines. Recommendations on optimal timing for antenatal treatments and the need for post-infancy booster vaccinations shaped vaccination strategies and helminth control programs. The research also emphasized the importance of maternal health as a determinant of child health outcomes, influencing public health policies at the local and national levels (24).

**Policy Citations**: Table 3 highlights the geographic distribution of policy citations referencing Malhotra’s research. Her work is cited in five policy documents across multiple countries and organizations, reflecting its global impact on policy and clinical guidance. Notably, two citations appear in United States. clinical guidance documents, underscoring its practical relevance to clinical decision-making. The remaining three citations originate from the European Union, France, and intergovernmental organizations, further demonstrating the broad applicability of Malhotra’s contributions.

**Table 3:** Policy citations of Malhotra’s research, by country*.

Source country	Policy document type		Grand total
	Clinical guidance	Publication	
EU		1	1
France		1	1
IGO		1	1
USA	2		2
Grand Total	2	3	5

*Retrieved from https://www.overton.io/ on December 1, 2024.

##### 3.2.3.3 Populations affected, geographical impact and research expansion

**Populations and Geographic Focus**: Malhotra’s research targeted economically challenged populations in Kenya’s Kwale District, specifically pregnant women and their infants, who face heightened risks from parasitic infections such as malaria and schistosomiasis. These infections often result in severe health challenges, including malnutrition and anemia, which disproportionately affect maternal and child health outcomes in low-resource settings. Kwale, a rural region along Kenya’s south coast, exemplifies the intersection of poverty and endemic disease, with challenges like inadequate access to healthcare, potable water, and sanitation creating an environment where vector-borne and parasitic diseases thrive (24). By focusing on this region, the study aimed to develop locally relevant and scalable interventions to improve health outcomes for mothers and infants. The findings underscored the critical need to address underlying factors such as sanitation, healthcare access, and nutrition to mitigate the impacts of parasitic diseases, providing an integrated approach to improving maternal and child health in low-resource environments.

**Focus on High-Risk Populations**: The populations studied were socially and economically challenged. The communities faced extreme poverty, with household incomes often below $25 per month. Educational attainment was low, with high illiteracy rates among adults, particularly women. These conditions limited the ability of individuals to access and understand healthcare services. Moreover, the prevalence of parasitic infections in these populations was alarmingly high, affecting 60–70% of pregnant women, with many suffering from co-infections. Malnutrition, anemia, and stunted growth exacerbated their already precarious condition. The patriarchal social structure in these communities added another layer of complexity, as decisions regarding healthcare were often made by male heads of households, requiring additional efforts to engage and educate families. Combined with social stigmas surrounding conditions like HIV, these factors deterred many women from seeking antenatal care, highlighting the critical need for community-based interventions (personal communication, November 2024).

**Research Expansion to Additional Populations**: Building on its success in Kwale, the research model has been adapted to other endemic regions, including Kenya’s South Coast (personal communication, November 2024). These expansions address similar challenges, such as parasitic infections, anemia, and malnutrition, with a growing focus on children’s health. The team continues to work in partnership with local governments and international organizations to develop scalable, context-specific interventions aimed at promoting sustainable improvements in health outcomes across regions affected by parasitic diseases.

##### 3.2.3.4 Advancements in public health

Improving access to care was the guiding principle of Malhotra’s research, which introduced low-cost, efficient diagnostic innovations such as the specimen-sparing bead assay for susceptible communities. These innovations mitigated obstacles to disease detection and treatment, particularly among populations where invasive procedures were either clinically impractical or met with cultural resistance.

A major strength of this initiative was its emphasis on capacity building. Training programs for local health workers, laboratory scientists, and researchers enhanced the delivery of healthcare services while establishing a foundation for long-term public health improvements. By empowering communities with the skills to address future health challenges, the project reduced reliance on external support and fostered sustainability (28).

The research also emphasized a community-centered model that combined education and preventive care. Local health workers engaged mothers in promoting practices like malaria prophylaxis and vaccination. This approach not only reduced infection prevalence but also equipped healthcare providers with the skills to independently manage and monitor parasitic infections (personal communication, November 2024). The success of this model highlights the transformative impact of community engagement in achieving sustainable health outcomes.

##### 3.2.3.5 Expanding the research impact

The research team is pursuing new directions to expand the impact of their work. Current efforts include studies on anemia and malnutrition among children, building on previous findings of stunting and growth deficiencies in the population. By examining the complex links between maternal health, infections, and childhood nutrition, and collaborating with local initiatives, the team integrates health improvement with environmental sustainability to address broader determinants of health. Proven interventions, such as maternal treatments and mass vaccination campaigns, are being scaled to other regions with similar needs. Through partnerships with local and international stakeholders, the team strives to translate research into actionable policies and programs that improve health outcomes.

#### 3.2.4 Case study 4. *Traumatic brain injury among the survivors of intimate partner violence (IPV),* Gunnur Karakurt, LMFT, PhD

##### 3.2.4.1 Utilization of the CTSC

Karakurt engaged with the CTSC through the KL2 Training Program, which played a pivotal role in supporting her career development. In addition to structured training, Karakurt leveraged a broad array of CTSC resources, including expert reviews to assess health outcomes in research instruments, editorial support to enhance the clarity of research documents, and guidance in the technological development of diagnostic tools. Furthermore, participation in CTSC-sponsored networking events facilitated valuable community support and fostered interdisciplinary collaborations.

##### 3.2.4.2 Societal benefits in TSBM categories

Karakurt’s research demonstrated societal benefits across several TSBM categories:

**Clinical and Medical Benefits**: Subtype Identification of IPV (29) and IPVDetect (30) are web-based applications that leverage clinical data and AI technology to improve the identification, understanding, and management of intimate partner violence (IPV), thereby supporting both individuals and professionals in addressing this critical social issue effectively.

The IPV Subtyping Tool is a questionnaire-based application that categorizes relationships into specific subtypes of IPV based on the responses provided. It utilizes statistical models developed from integrated datasets, which include clinical data and community observations from both healthy and unhealthy relationships. The tool identifies six subtypes of relationships, ranging from emotionally abusive to physically and sexually abusive scenarios. This categorization helps individuals in troubled relationships understand the severity and nature of their situation (31). For individuals, it delivers a scientific assessment of relationship health, helping them recognize the severity of their situation. Therapists benefit from precise subtype identification, enabling more targeted therapeutic approaches. The tool also connects users to appropriate resources, such as information about shelters or counseling, based on their results (personal communication, November, 2024).

Designed during the pandemic to address rising IPV cases, IPVDetect uses AI-driven text analysis to identify abusive patterns in user-generated content. It screens user-generated content (e.g., personal anecdotes) for indicators of IPV. By analyzing the language used, the tool highlights phrases and sentences that suggest different forms of abuse—physical, emotional, or sexual (32). It provides individuals with immediate feedback on abusive dynamics, fostering self-awareness. For clinicians, it offers a rapid assessment mechanism to determine the type of abuse and severity, enabling more focused and informed clinical interventions. Additionally, it has potential applications in legal and educational contexts, enhancing understanding of IPV nuances in courtrooms and training environments.

**Community and Public Health Benefits**: The research enhanced community health services by developing diagnostic tools for IPV survivors. Notably, potential collaborations with healthcare systems such as Epic EHR may integrate these tools into broader health IT infrastructures, facilitating risk assessments and information delivery for IPV survivors.

**Policy and Legislative Benefits**: Karakurt’s research has had tangible impacts on policy, notably in the Philippines where it influenced the legal treatment of emotional abuse (33). Furthermore, the research was presented in a United States. Congressional hearing, highlighting its relevance and applicability in shaping public policy and legal frameworks (34, 35).

**Policy Citations**: Table 4 highlights the global influence of Karakurt’s research, cited in 28 policy documents across 9 countries and IGOs, demonstrating its critical role in research-informed policymaking. The distribution of citations across countries and document types underscores the relevance of Karakurt’s research. Its integration into clinical guidance, legal frameworks, academic publications, and working papers highlights its interdisciplinary nature and its ability to inform complex, cross-sectoral challenges.

**Table 4:** Policy citations of Karakurt’s research, by country*.

Source country	Policy document type					Grand total
	Clinical guidance	Legal documents	Publication	Scholarly article	Working paper	
Australia			3			3
Canada			1	1		2
Colombia			1			1
Finland			1			1
IGO			5		2	7
Norway	1					1
Peru			2			2
Philippines			1			1
Sweden	1	1	2			4
USA			6			6
Grand Total	2	1	22	1	2	28

*Retrieved from https://www.overton.io/ on December 1, 2024.

##### 3.2.4.3 Populations affected, geographical impact and research expansion

**Populations**: Karakurt’s research on IPV responds to the critical gaps in resilience-informed care for affected populations facing persistent access challenges. The study examined survivors receiving services at a Rape Crisis Center located in an urban area of the Midwestern United States, with participants representing high-risk groups (36). These populations intersecting challenges such as social and economic hardship, cultural stigma, and inadequate access to tailored support. By integrating a range of perspectives across educational and economic backgrounds, the study highlights the critical need for user-friendly interventions (personal communication, November 2024; 36).

**Focus on High-Risk Populations**: Karakurt’s research centers on populations experiencing compounded challenges, with a particular focus on the multifaceted impact of IPV. Children exposed to violence are at an elevated risk for developmental delays and long-term psychological health challenges stemming from Adverse Childhood Experiences (ACEs). IPV-related injuries frequently result in traumatic brain injury (TBI), with 60 to 92% of female survivors reporting facial, head, or neck strangulation injuries (37, 38). These findings underscore IPV as a pressing public health concern with enduring, cross-generational consequences. Karakurt’s work further highlights the unique challenges faced by high-risk groups and calls for community-centered approaches that are responsive to the needs and lived experiences of all communities (personal communication, November 2024).

**Expansion to Additional Populations**: Karakurt is expanding her research to include IPV prevention, with a focus on public education around emotional abuse, healthy relationships, and available support systems. For example, she contributes as an advisor to the UN Women Implementation Guidance (39). By empowering individuals with knowledge about the early warning signs and preventive strategies, Karakurt aims to curb IPV before it escalates, which aligns with foundational public health strategies of disease prevention and health promotion.

##### 3.2.4.4 Advancements in public health

Improving health outcomes is at the core of Karakurt’s work, reflecting her commitment to developing practical, data-driven solutions for all populations. Acknowledging the structural challenges that limit access to care for under-resourced individuals, she has deliberately designed her research methodologies and intervention tools to be both easy to use and cost-free. This intentional approach ensures that no individual is denied help due to social and economic constraints. As Karakurt notes, “*Intimate partner violence is considered a public health problem, and there is an urgent need for scalable … interventions*.” Her emphasis on ease of use not only addresses the immediate needs of survivors but also contributes to broader system improvements. By expanding access to care, increasing public awareness, and equipping individuals with actionable resources, Karakurt’s work contributes meaningfully to reducing both the incidence and societal burden of intimate partner violence.

##### 3.2.4.5 Expanding the research impact

Karakurt plans to continue exploring IPV through funding for advanced statistical tool development and national health data integration, aiming to address prevention and early intervention more effectively. The research goals include enhancing the access and functionality of existing tools and extending their reach to impact a broader demographic nationally and internationally.

#### 3.2.5 Case study 5. *Non-endoscopic screening for Barrett’s esophagus*, Amitabh Chak, MD

##### 3.2.5.1 Utilization of the CTSC

Chak utilized the CTSC resources across multiple projects related to Barrett’s Esophagus (BE). In October 2016, he received the Core Utilization Pilot grant to use the FDA Guidance Core, facilitating crucial early development of non-endoscopic screening technologies. Chak further accessed the CTSC in January 2023 to support a multicenter randomized controlled trial, SURVENT, which compares surveillance versus endoscopic therapy for BE with low-grade dysplasia. In October 2024, Chak used the University Hospitals REDCap to support a project aimed at detecting BE in patients without symptoms of gastroesophageal reflux disease (GERD), broadening the applicability of his work.

##### 3.2.5.2 Societal benefits in TSBM categories

Chak’s research has shown profound societal benefits across several TSBM categories:

**Clinical and Medical Benefits**: The EsoCheck device and EsoGuard lab test have revolutionized the diagnosis of esophageal conditions, offering a non-invasive, highly accurate alternative to traditional endoscopy. Recognized in guidelines by the American College of Gastroenterology (ACG) and American Gastroenterological Association (AGA), and awarded Breakthrough Device Designation by the FDA, these technologies are endorsed as transformative tools in gastroenterology (40).

EsoGuard, a laboratory-developed test, analyzes 2 methylated DNA biomarkers with remarkable precision. In a pivotal NIH-sponsored clinical trial involving 86 patients, the test demonstrated over 90% sensitivity and specificity for detecting BE (41). A follow-up study confirmed these results in 322 patients (42). Beyond BE, EsoGuard detects a range of esophageal conditions, including dysplastic BE and esophageal adenocarcinoma, making it a versatile tool for early detection and prevention.

EsoCheck is the only minimally invasive, nonendoscopic method for detecting BE that is approved for use in the US. Its non-invasive approach reduces the need for endoscopy, aligning with modern, patient-centered diagnostic practices.

**Community and Public Health**: The deployment of these innovations in rural areas and through mobile health units addresses healthcare access, particularly in rural areas such as Wayne County, Ohio, and in health fairs across Florida.

**Economic Benefits**: The commercialization of these technologies through Lucid Diagnostics, co-founded by Chak and colleagues, has translated research into practical applications, leading to patented innovations and the creation of economic value through new healthcare products and services (43).

**Policy Citations**: As displayed in Table 5, Chak’s research has influenced 60 policy citations across 11 countries, including 50 that were cited in clinical guidance documents. The predominance of clinical guidance citations (83%) highlights the research’s practical impact in shaping healthcare standards.

**Table 5:** Policy citations to Chak’s research, by country*.

Source country	Policy document type		Grand total
	Clinical guidance	Publication	
Australia		2	2
Canada	1		1
EU		1	1
Finland	1		1
Germany	12	1	13
Italy	1		1
Netherlands		1	1
Spain		1	1
Turkey		1	1
UK	12		12
USA	23	3	26
Grand Total	50	10	60

*Retrieved from https://www.overton.io/ on December 1, 2024.

##### 3.2.5.3 Populations affected, geographical impact and research expansion

**High-Risk Groups and Screening Needs**: EsoGuard and EsoCheck are used to screen patients with GERD, non-dysplastic and dysplastic BE, and early-stage adenocarcinoma of the esophagus or gastroesophageal junction. BE, the only known precursor to esophageal adenocarcinoma (EAC), is most common in individuals with chronic GERD, yet the low annual progression rate to cancer requires cost-effective and scalable monitoring solutions (44).

**Geographic Impact**: Chak’s research addresses improved health outcomes for all people by deploying EsoGuard and EsoCheck in geographically isolated regions. Screening programs in rural Ohio counties and mobile health fairs in Florida bridge gaps in healthcare access, providing life-saving diagnostics to populations lacking proximity to specialized care (43). Mobile health initiatives further extend the reach of these technologies, ensuring that economically challenged communities benefit from early detection services.

**Registry-Based Research**: Registries such as the Prospective REView of Esophageal Precancer DetectioN in AT-Risk Patients (PREVENT) Registry and the CLinical Utility of EsoGuard (CLUE) study collect real-world data on the performance of these technologies (45, 46). Additionally, the PREVENT-FF Registry focuses on high-risk firefighter populations (47). These registries provide essential insights into the use of EsoGuard and EsoCheck in high-risk groups, enabling the optimization of screening protocols and expanding their utility (43).

**Focus on High-Risk Populations**: A key element of Chak’s work is its emphasis on high-risk groups, including rural populations and high-risk occupations. Initiatives in Wayne County, Ohio, and Florida provide essential screening services to individuals who may lack healthcare access (48, 49). Firefighters, who face a 62% higher risk of esophageal cancer due to occupational carcinogen exposure, are a priority group in this research (47). Targeted screening programs not only offer early detection but also address the unique healthcare needs of those serving their communities, demonstrating the importance of tailored healthcare solutions (50).

**Expanding Research to Broader Populations**: Chak’s research continues to broaden, extending to former NFL players and other populations via #CheckYourFoodTube events, which raise awareness and improve access to life-saving diagnostics (51).

##### 3.2.5.4 Advancements in public health

The research on BE has improved public health by introducing innovative, non-invasive screening methods. These advancements make detection more convenient, less intimidating, and more practical for high-risk populations.

The introduction of EsoCheck (EC) with EsoGuard (EG) has transformed BE screening practices. This approach reduces the discomfort, risk, and logistical challenges associated with traditional endoscopy, thereby increasing patient willingness to undergo screening (40, 41).

The portability and ease of use of the EC/EG device have enabled its deployment in mobile health units staffed by nurse practitioners. These units serve rural communities, where access to specialized care is often limited. By bringing screening directly to these populations, this research bridges critical gaps in healthcare access, fosters early detection and intervention, and improves health outcomes.

##### 3.2.5.5 Expanding the research impact

To address the rising burden of esophageal cancer, Chak proposes a comprehensive strategy to expand access and enhance early detection. Central to this effort is advocating for health insurance coverage of EC/EG screening tests. Insurance support is crucial for improving affordability and access, especially for high-risk populations. Removing cost-related obstacles is essential to expanding access to life-saving diagnostic technologies.

Expanding screening access is another critical objective. By collaborating with health centers nationwide, Chak aims to establish a robust network of advanced screening programs. This expansion will prioritize high-risk groups, including firefighters, middle-aged individuals with GERD, former NFL players, people over 50, individuals with elevated body weight, and smokers. Targeting these populations enables efficient resource use and improved outcomes.

An urgent challenge is the “silent risk” of EAC—cases where patients develop cancer without presenting GERD symptoms. These asymptomatic cases often go undetected until the disease is advanced, reducing the chances of successful treatment. To address this gap, Chak has submitted grant proposals to support research and interventions targeting high-risk individuals who lack GERD symptoms, aiming to improve early detection in these cases.

Additionally, advancing biomarker research is pivotal to refining screening strategies. By developing and validating biomarkers that identify individuals at the highest risk of EAC, screening can be tailored to those most likely to benefit. This precision approach will enhance the impact of early detection efforts.

#### 3.2.6 Case study 6. *Hemoglobin electrophoresis Biochip for newborns*, Umut a. Gurkan, PhD

##### 3.2.6.1 Utilization of the CTSC

Gurkan and his team at CWRU leveraged the CTSC’s funding, infrastructure, and logistical support to advance groundbreaking research from concept to innovation. The CTSC provided critical pilot funding for key projects, including the foundational 2014 *“Hemoglobin Electrophoresis Biochip for Newborns”* project, which established scalable diagnostic technologies, and subsequent studies such as the 2020 *“Microfluidic Blood Cell Adhesion Test for Anti-Adhesive Therapies”* and the 2021 *“Microfluidic Blood–Brain-Barrier for Modeling Permeability during Health and Disease States.”* Further support came through a CTSC Research consultation, aiding the development of the SMART (Sickle, Malaria, Anemia Rapid Test) device, and the facilitation of the 2020 Doris Duke Clinical Foundation Data Sharing Project, contributing to a national sickle cell disease database. Beyond funding, the CTSC streamlined operational processes like IRB approvals, funded personnel, and fostered interdisciplinary collaboration, enabling Gurkan’s team to overcome early-stage research challenges and propel these projects toward impactful outcomes.

##### 3.2.6.2 Societal benefits in TSBM categories

Gurkan’s research has demonstrated societal impact across multiple TSBM categories:

**Clinical and Medical Benefits**: The Gazelle platform transforms diagnostics with portable, affordable point-of-care testing for conditions like sickle cell disease, thalassemia, anemia, COVID-19, and hemoglobin disorders. By bridging diagnostic gaps in economically challenged regions, Gazelle enables global access to accurate testing (52–59).

- Clinical microfluidic assays, developed through a 2020 Pilot Award, are now used as biomarker endpoints in pharmaceutical clinical trials and distributed by BioChip Labs Inc. (60).
- The ClotChip device, developed with Dr. Pedram Mohseni’s team, assesses whole-blood coagulation and addresses critical needs in areas such as congenital disorders, anticoagulant therapies, and preoperative evaluations (61). Awarded FDA Breakthrough Device Designation in March 2020, ClotChip is undergoing clinical trials to further expand its impact (62).

**Community and Public Health Benefits**: Gurkan’s research targeted global populations, including groups in India and rural communities in Sub-Saharan Africa. By integrating diagnostic tools into local healthcare workflows, such as vaccination programs and primary care visits, the team advanced healthcare delivery.

**Economic Benefits**: The HemeChip, an earlier version of the Gazelle platform, was licensed to Hemex Health in 2016, leading to its global commercialization. Deployed in over 42 countries, the device strengthens local healthcare systems and creates sustainable diagnostic infrastructure (63). With 49 US patents and/or patent applications related to diagnostic technologies, including the Gazelle, clinical microfluidic assays, and ClotChip devices, Gurkan has built a strong intellectual property foundation that ensures scalability and global impact.

**Policy and Legislative Benefits**: Persistent promotion led to adding hemoglobin electrophoresis to WHO guidelines for universal sickle cell disease screening, a major step in addressing global health. Regulatory approvals of the Gazelle platform in India, Europe, and Africa underscore the technology’s adaptability (64).

##### 3.2.6.3 Populations affected, geographical impact and research expansion

This research has transformed healthcare for groups affected by hemoglobin disorders, anemia, and other infectious diseases worldwide (personal communication, December 2024).

**Populations Affected**: The research has targeted many groups facing critical health challenges:

- **Sickle Cell Disease (SCD) Patients**: In malaria-endemic regions like Sub-Saharan Africa and India, SCD is often underdiagnosed or misdiagnosed, exacerbating health outcomes.
- **Thalassemia Patients**: Widespread in the Middle East, Europe, Southeast Asia, Turkey, and India, thalassemia impacts rural and general populations.
- **Anemia Patients**: Anemia affects individuals with nutritional deficiencies, parasitic infections, or complications from SCD and thalassemia. Women and children are disproportionately affected due to malnutrition and limited healthcare.
- **Children and Newborns**: In regions without newborn screening programs, many infants born with SCD remain undiagnosed, leading to high mortality rates.
- **Undiagnosed Patients**: Many individuals with genetic hemoglobin variants remain undiagnosed due to a lack of affordable diagnostic technologies.

**Geographic Scope**: The Gazelle diagnostic platform has been deployed in over 42 countries, addressing diagnostic gaps worldwide (65).

- **Sub-Saharan Africa**: A significant focus of the research is on countries like Nigeria, Ghana, where rural and tribal populations face the dual burden of malaria and sickle cell disease due to limited access to healthcare.
- **India**: Tribal populations and rural communities with high prevalence of sickle cell disease and thalassemia are key targets. India supports national programs for hemoglobinopathies using tools developed through this research.
- **Middle East and Turkey**: Thalassemia affects both rural populations and the general population, demonstrating the disease’s widespread impact in these regions.
- **Southeast Asia and South America**: Populations in these regions benefit from scalable diagnostic tools that address hemoglobin disorders, anemia, and other health challenges.
- **Developed Countries**: In the United States, Canada, and Europe, the research supports public health programs for diagnostic gaps among hemoglobin variants.

**Focus on High-Risk Populations**: The research targeted populations that are disproportionately affected due to social, economic, and other obstacles (personal communication, December 2024):

- **Tribal Populations**: Limited access to mainstream healthcare among tribal communities in Africa and India contributes to elevated rates of diseases such as sickle cell disease and thalassemia.
- **Rural Communities**: Residents of remote areas lack access to healthcare infrastructure, leading to higher rates of undiagnosed conditions.
- **Women and Children**: Women bear the burden of anemia due to nutritional deficiencies and reproductive health issues, while children in low-resource settings often face delays in diagnosis and treatment of blood disorders.
- **Economic and Geographic Obstacles**: Populations in sub-Saharan Africa and India frequently encounter obstacles such as the high cost of diagnostics and the absence of systematic screening programs.
- **Contextual and Structural Limitations**: In some regions, local practices or logistical challenges (e.g., short hospital stays after birth) hinder early screening efforts. In rural Africa, integrating screening into vaccination programs was necessary to reach broader populations.
- **Middle Eastern and European Contexts**: In regions like Turkey, refugees and displaced individuals face disproportionate burdens of thalassemia and other conditions due to sometimes limited healthcare access.

##### 3.2.6.4 Advancements in public health

Gurkan’s vision is encapsulated in his statement:

*“I wish that everyone living with sickle cell disease had access to the same quality of care, diagnostic technologies, and curative treatments anywhere in the world. We’re working on making these diagnostic technologies more affordable and more available to everyone. We don’t want dust, temperature, dirt, cost, or complexity to be a [obstacle] for using technology to fight disease”* (66).

This vision underpins his pioneering efforts to improve public health globally. Gurkan’s research addresses widespread healthcare challenges through transformative approaches:

- **Available Diagnostics**: By developing cost-effective, portable diagnostic devices like the Gazelle platform, he has made screening and diagnosis feasible even in resource-constrained environments. These devices overcome obstacles such as cost, environmental challenges, and complexity, making them usable in remote regions.
- **Prioritizing High-Risk Populations**: Rural and native communities, particularly in Africa and India, are at the center of his intervention efforts. This prioritization ensures that populations with the highest disease burden and least access to care directly benefit from these innovations.
- **Culturally Adapted Solutions**: Diagnostic tools are tailored to local contexts. For instance, in regions where newborn hospital stays are brief, diagnostics were integrated into routine vaccination programs, enhancing access without disrupting existing healthcare workflows.

Furthermore, Gurkan’s work has significantly improved public health outcomes through targeted interventions:

- **Integration with Public Health Programs**: In Ghana, diagnostics were incorporated into newborn and vaccination-based screening programs, enabling early detection and treatment of sickle cell disease. In India, his research supported national programs addressing both sickle cell disease and thalassemia.
- **Improved Diagnostic Accuracy**: In malaria-endemic regions, where sickle cell disease is often misdiagnosed as malaria or another infectious disease, Gurkan’s technologies have transformed diagnostic accuracy. This ensures appropriate treatments are administered, reducing morbidity and mortality rates.
- **Strengthening Health Systems**: By addressing diagnostic gaps in low- and middle-income countries, his research has bolstered public health infrastructures, supporting the sustainable delivery of care to all populations.
- **Recognition by WHO**: In 2019, the WHO listed hemoglobin electrophoresis as an essential *in vitro* diagnostic test for SCD and sickle cell trait in low- and middle-income countries (64).

##### 3.2.6.5 Expanding the research impact

Gurkan’s research continues to evolve with a clear focus on tackling urgent healthcare challenges and amplifying the global impact of diagnostic technologies. Key initiatives driving this progress include:

- **Regulatory Approvals**: Securing FDA approval for diagnostic devices is a critical milestone, ensuring compliance with stringent regulatory standards and clinical requirements. This will enable widespread adoption in the US healthcare market, enhance international credibility, and unlock global market opportunities.
- **Development of Non-Invasive Diagnostics**: The team prioritizes the development of non-invasive methods to (a) eliminate the need for blood samples, addressing logistical and cultural obstacles associated with invasive testing; (b) increase access for rural and low-resource populations; (c) enhance patient experience, particularly for children and those apprehensive about traditional testing.
- **Expanded Diagnostic Capabilities**: The platform is evolving into a versatile tool to combat multiple health challenges:

o **Blood Disorders**: Sustained advancements in sickle cell disease and thalassemia diagnostics remain central.
o **Nutritional Deficiencies**: The addition of ferritin testing enables comprehensive anemia diagnostics, addressing critical nutritional issues.
o **Chronic Diseases**: Expanding capabilities to include diabetes testing (55) demonstrates the platform’s commitment to addressing the growing burden of chronic diseases.
o **Oral Cancer Screening**: Introducing biomarker-based diagnostics for oral cancer (67), particularly targeting high-risk populations in regions like India where tobacco-related cancers are widespread.

- **Data Integration and Advanced Analytics**: Advanced analytics drive future breakthroughs:

o Contributions to national disease databases, such as sickle cell anemia, enhance research and care.
o AI and data mining uncover insights, improving diagnostic algorithms and public health strategies (57, 68–70).
o Refinements in diagnostic accuracy and efficiency strengthen the platform’s impact.

- **Customization for Local Contexts**: To ensure effective adoption across multiple healthcare systems, the team tailors diagnostics to regional needs:

o Adapting workflows to align with local practices and resources.
o Engaging healthcare workers and patients to develop user-friendly, appropriate solutions (71, 72).

## 4 Discussion

### 4.1 Addressing methodological challenges in translational research

Although the TSBM provides a robust framework for capturing the societal benefits of translational research, the use of single-method study designs, such as surveys, can quantify the TSBM benefits but with limitations to fully explain the value of translational research. This study addresses critical methodological challenges in evaluating the outcomes of translational research. Traditional survey methods often fall short in capturing the multifaceted and dynamic nature of translational research. Issues such as outdated data, participant survey fatigue, and the undervaluation of surveys pose significant obstacles to collecting actionable and comprehensive insights. These limitations underscore the necessity of adopting complementary methodologies that can capture the breadth and depth of translational research impacts.

To overcome these challenges, this study employs a mixed-methods approach that integrates various tools designed to enhance the evaluation of translational research outcomes. First, supporting documentation—such as CVs, publications, and other tangible outputs—was incorporated to validate and enrich the survey data. These materials offer concrete evidence of scholarly productivity and societal benefit, providing a more complete picture of how research translates into real-world impact. By grounding the analysis in verified outputs, this approach enhances both the robustness and credibility of the evaluation.

In addition, qualitative interviews provide in-depth narratives that offer critical insights into the lived experiences of researchers and the pathways through which their work advances public health. These interviews move beyond surface-level outcomes to reveal the nuanced, context-dependent processes that drive successful translational efforts. They help illuminate how discoveries move from academic settings into policy, practice, and community benefit.

An innovative component of this study is the use of research databases including Overton (3) and Dimensions (4) to identify and analyze global policy impacts of translational research. By generating policy citation data, these tools provide a comprehensive lens to evaluate the societal relevance and international reach of translational science. They complement qualitative and survey data by documenting the influence of research on policy and legislative decisions, underscoring the broader implications of translational efforts on public health.

Together, these integrated methods demonstrate the value of a mixed-methods approach in evaluating the translational impact of research supported by the CTSC. The combination of survey data, publications, qualitative interviews, and policy citations enables a nuanced understanding of how research contributes to tangible societal benefits. This methodological framework not only addresses critical gaps in traditional evaluation practices but also offers a replicable model for future translational impact evaluations. Importantly, by leveraging multiple methods and data sources, the study advances a comprehensive and adaptable evaluation paradigm that aligns with the complex and dynamic nature of translational science.

### 4.2 Advancing public health through translational science: insights from six case studies

#### 4.2.1 Advancing health outcomes through targeted interventions

A consistent theme across all six case studies is the focus on addressing healthcare challenges through tailored, innovative interventions for low-resource and high-risk populations. For example, Bolen’s work in Ohio targeted Medicaid-covered individuals disproportionately affected by chronic conditions such as hypertension and diabetes. By employing a positive deviance model in primary care, her interventions significantly improved hypertension control and overall health outcomes across multiple affected groups. Similarly, Malhotra’s maternal health research offer valuable evidence for optimizing immunization schedules and maternal health interventions. The development of minimally invasive, cost-effective diagnostic technologies, like multiplex bead assays, has broad applicability for high-risk US communities where healthcare access and early disease detection remain challenges. Additionally, her community-engaged, capacity-building model also underscores the effectiveness of grassroots health education and decentralized care, offering scalable strategies to improve maternal-child health outcomes among economically challenged populations.

#### 4.2.2 Transformative innovations in technology and public health

Innovation was the cornerstone of these projects, propelling breakthroughs in diagnosis, treatment, and prevention. Chak’s non-invasive screening tools, EsoCheck and EsoGuard, have transformed healthcare delivery for BE and esophageal cancer. By offering accurate, affordable, and convenient alternatives to traditional endoscopy, these technologies have expanded screening access to high-risk populations, including those in rural areas. Similarly, Gurkan’s Gazelle diagnostic platform has redefined point-of-care testing for conditions like sickle cell disease, thalassemia, and anemia. Compact and affordable, the platform delivers rapid, reliable diagnostics in resource-limited settings, reducing dependency on centralized laboratories and facilitating early treatment interventions.

#### 4.2.3 Policy and systems-level impact

These projects demonstrated the far-reaching impact of translational science, driving systemwide changes in policies and healthcare systems while addressing complex health challenges. Bolen’s research played a pivotal role in shaping Medicaid reforms, expanding access to preventive care for diabetes and hypertension, and removing obstacles to diabetes technology. Her contributions exemplify how research can directly influence state and national policies, creating better healthcare systems for all people. Similarly, Malhotra’s maternal health project integrated research findings into Kenyan health strategies by collaborating with local authorities, resulting in sustainable improvements to vaccination schedules and parasitic disease control programs.

The global policy citations of these researchers’ findings further highlights their broad influence. Their work informed many policy documents, including clinical guidelines, legal frameworks, and scholarly analyses. This variety underscores the adaptability and relevance of their research across different policymaking contexts.

Collectively, these efforts illustrate the transformative power of aligning research priorities with public health needs. By bridging disciplines and fostering collaboration, these projects created scalable, research-informed interventions with long-term, global impact. The success of these researchers underscores the critical role of translational science in shaping policies that improve health outcomes, demonstrating how robust science and interdisciplinary approaches can drive sustainable change.

#### 4.2.4 Implications for translational science and public health

Collectively, these case studies highlight four core principles that underpin effective translational science and its public health impact:

- **Prioritize Effective Innovations**: Translational efforts must directly address challenges faced by high-risk populations. Malhotra’s maternal health initiative used tailored diagnostics and education to tackle parasitic infections and anemia, creating lasting improvements in maternal and infant health. These approaches help close care gaps while fostering trust and engagement within economically challenged communities.
- **Ensure Scalability and Sustainability**: The success of translational science hinges on its ability to produce models that are both scalable and sustainable. The case studies highlight how interventions can be effectively adapted for multiple contexts while maintaining their core impact. Gurkan’s Gazelle diagnostic platform, originally designed for sickle cell disease, now addresses multiple conditions and is deployed in over 42 countries, demonstrating its scalability. Sustainability complements scalability by embedding community ownership and capacity-building into intervention frameworks. Malhotra’s project trained local healthcare providers, ensuring the longevity of diagnostics and educational initiatives. Such approaches guarantee that innovations outlive their research phases, delivering long-term benefits.
- **Align with Policy to Maximize Impact**: A critical takeaway from these case studies is the necessity of aligning research objectives with public policy to maximize impact. Bolen’s work informed Medicaid reforms, expanding access to preventive care and life-saving technologies, while Knighton’s hygiene education initiatives shaped state-level COVID-19 policies. When research influences legislation, localized interventions evolve into universal, transformative change.
- **Foster Collaborative Approaches**: The case studies highlight the critical role of interdisciplinary collaboration and community engagement in creating impactful and sustainable health solutions. Translational science thrives with broad collaboration from many areas of expertise—researchers, practitioners, policymakers, and community stakeholders—converges to address complex health challenges. Malhotra’s maternal health initiative exemplifies this by partnering with local health authorities and community leaders to ensure relevant and widely accepted interventions. Engaging communities as co-creators not only builds trust but also fosters ownership, ensuring solutions are tailored to local needs and sustainable over time. This collaborative model drives lasting impact and empowers communities to embrace and sustain meaningful change.

### 4.3 Challenges and facilitators in CTSC-supported research

The six case studies presented here reveal a shared landscape of opportunities and obstacles faced by investigators engaged in translational, community-based, and technology-driven research initiatives supported by the CTSC. Despite the wide-ranging focus areas—including infection prevention, IPV, global diagnostics, and early-stage medical technologies—these projects shared common struggles that reflect broader universal challenges within the translational research ecosystem. At the same time, they demonstrate how strategic organizational support, coupled with community-rooted approaches, can convert local challenges into scalable innovations.

#### 4.3.1 Persistent challenges in translational and community-engaged research

Resource constraints emerged as a foundational obstacle across nearly every initiative. Investigators frequently cited insufficient, short-term funding as a limiting factor, especially in efforts requiring longitudinal engagement with communities or extended periods to demonstrate health outcomes. Bolen and Malhotra, for instance, described how the episodic nature of grant funding clashed with the sustained effort needed to build trust, infrastructure, and evidence in high-risk populations. Similarly, Knighton’s public health intervention struggled to scale due to the high upfront costs of technology deployment and limited avenues for sustained financial support.

Administrative and regulatory requirements posed significant obstacles, especially for projects situated at the intersection of clinical innovation and public health. Complex processes—such as coordinating across multiple IRBs, obtaining FDA approvals, and managing subcontracts—often caused delays and placed additional strain on already limited resources. Karakurt and Chak’s experiences underscore the sustained time and effort required to conduct ethically sensitive or high-risk research. These challenges disproportionately impact early-career researchers and community-based organizations, which often lack the administrative infrastructure needed to manage such complexity.

In parallel, investigators encountered deep-seated obstacles to access and engagement. Mistrust of research institutions—whether due to historical abuses, cultural beliefs, or lack of representation—was a recurrent theme, particularly in projects working with specific subgroups or rural populations. Malhotra’s work with traditional birth attendants and community leaders and Karakurt’s engagement with IPV survivors reveal the fragility of researcher-community relationships when not rooted in humility and sustained presence. Moreover, logistical issues such as transportation, electricity, and internet access in global or rural settings amplified these engagement challenges, threatening both recruitment and retention.

Researchers working in emotionally demanding fields such as IPV often encountered less visible, yet deeply impactful, challenges—including secondary traumatic stress and emotional exhaustion. These burdens, which extend beyond measurable scientific outputs, underscore the need to embed emotional resilience as a foundational element of the research environment.

#### 4.3.2 Key facilitators of research success

Several key facilitators emerged that enabled investigators not only to overcome these challenges but to generate meaningful, lasting impact.

First, the CTSC itself functioned as a backbone of support, offering more than just funding. Across cases, the CTSC provided essential mentorship, infrastructure, pilot grants, and access to cross-disciplinary expertise. For many investigators, particularly those in the early stages of their careers, this ecosystem of support was catalytic—transforming isolated efforts into well-resourced, collaborative programs with the credibility to secure additional investment. Programs such as the KL2 training grant equipped researchers with the technical, ethical, and collaborative skills required to navigate complex research environments.

Equally critical was the commitment to community engagement and capacity building. The most effective projects invested in the long game: building trust, co-designing interventions, and empowering community members as co-investigators rather than passive participants. Malhotra’s use of community health workers and Bolen’s participatory research workshops not only enhanced research rigor and relevance but also laid the groundwork for sustainable change. These partnerships shifted power toward the community, increasing project legitimacy and fostering mutual accountability.

Strategic, cross-sector collaborations further amplified reach and resilience. Partnerships with health systems, philanthropic foundations, local leaders, and global organizations provided crucial credibility and operational support. For example, Knighton’s alignment with foundations enabled deeper community penetration during the pandemic, while international diagnostic projects leveraged global partnerships to overcome regulatory and cultural challenges across healthcare systems.

Another powerful enabler was the emphasis on adaptive, user-centered design. Investigators who embedded iterative feedback loops—whether from community members, healthcare providers, or policymakers—were able to refine tools and interventions to better fit real-world needs. This responsiveness not only improved adoption and engagement but also allowed for scalable, context-sensitive solutions.

Finally, small-scale pilot funding and seed grants played an outsized role in transforming early ideas into fundable, high-impact initiatives. These initial investments provided more than capital—they served as proof-of-concept platforms where investigators could test feasibility, generate preliminary data, and build stakeholder trust. The strategic deployment of pilot projects allowed for risk mitigation while creating momentum for larger-scale implementation and dissemination.

#### 4.3.3 Implications for CTSAs and translational science

These findings suggest that the most transformative translational research occurs not in isolation, but at the intersection of organizational infrastructure, community wisdom, and adaptive innovation. The CTSC’s role in these success stories was not merely transactional—it was transformational. By providing flexible support mechanisms, convening stakeholders, and fostering a culture of mentorship and collaboration, the CTSC created the conditions for innovation to take root in often challenging environments.

Yet, the work also illuminates persistent challenges in the translational research ecosystem. From inflexible funding timelines to burdensome administrative processes and limited emotional support for investigators, these difficulties must be addressed if translational science is to fulfill its promise of improving health for all.

These case studies offer not only insight but a blueprint—a roadmap for how organizational support, when aligned with local leadership and responsive innovation, can overcome universal challenges and catalyze meaningful change. They remind us that translational science, at its best, is not merely about moving discoveries from bench to bedside, but about bridging worlds: connecting knowledge to need, innovation to impartiality, and research into real life it seeks to serve.

## Data Availability

The datasets presented in this article are not readily available because it is private evaluation data from the Clinical and Translational Science Collaborative of Northern Ohio. Requests to access the datasets should be directed to clara.pelfrey@case.edu.
