# Supplementary material for: Demonstrating public health impacts of translational science at the clinical and translational science collaborative (CTSC) of northern Ohio: a mixed-methods approach using the translational science benefits model
Source: Front Public Health. 2025 Aug 13;13:1560751. doi: 10.3389/fpubh.2025.1560751 (PMC12380759; doi:10.3389/fpubh.2025.1560751)
Supplement: Supplementary file 1 [file Table_1.docx]

Supplementary Material

# Appendix

Supplementary Material should be uploaded separately on submission. Please include any supplementary data, figures and/or tables.

## Appendix A: TSBM Survey Instrument

| **Translational Science Benefits from CTSC Pilot Research**  Pilot Grant Research Title:  TRANSLATIONAL SCIENCE BENEFITS Checklist Use this checklist to track the CLINICAL, COMMUNITY, ECONOMIC, & POLICY benefits of your work.  Many research projects will have clinical and translational research benefits-or indicators-in multiple domains, as noted in the Translational Science Benefits Model (TSBM). The indicators are specific, observable, and are each at least potentially measurable.  For each indicator you select, please choose EITHER demonstrated (verifiable) or potential (highly likely), but not both.  TIPS   - Demonstrated benefits are those that have been observed and are verifiable; potential benefits are those logically expected with moderate to high confidence. - It is not expected that you have demonstrated or potential benefits in all domains.   DIRECTIONS: In the sections below, please indicate any translational science benefits that resulted from your CTSC Pilot Grant research. If demonstrated, please provide verifiable evidence.  (Please Note: It is NOT expected that you will have benefits in all domains)  **Clinical & Medical Benefits**  *CLINICAL & MEDICAL BENEFITS - Procedures and Guidelines*  Diagnostic procedures - Methods and techniques performed to diagnose disease, disorders, or conditions.  Demonstrated/Potential (If demonstrated, please provide verifiable evidence.)  Investigative procedures - Research methods used in pre-clinical, clinical, and other scientific studies. Demonstrated/Potential (If demonstrated, please provide verifiable evidence.)  Guidelines - Formal recommendations or principles to assist with patient care for specific clinical circumstances. Demonstrated/Potential (If demonstrated, please provide verifiable evidence.)  Therapeutic procedures - Methods & techniques that pertain to interventions, treatment, or prevention of diseases, disorders or conditions. Demonstrated/Potential (If demonstrated, please provide verifiable evidence.)  *CLINICAL & MEDICAL BENEFITS - Tools & Products*  Biological factors & products - Biological substances used to indicate, diagnose, prevent, or treat diseases or medical conditions. Demonstrated/Potential (If demonstrated, please provide verifiable evidence.)  Biomedical technology - Technology applications for solving medical problems. Demonstrated/Potential (If demonstrated, please provide verifiable evidence.)  Drugs - Pharmaceutical products for human or veterinary use. Demonstrated/Potential (If demonstrated, please provide verifiable evidence.)  Equipment & supplies - Apparatus, instruments, and materials for diagnostic, surgical, therapeutic, and scientific procedures. Demonstrated/Potential (If demonstrated, please provide verifiable evidence.)  Software technologies - Computer programs or software installed on mobile or other electronic devices. Demonstrated/Potential (If demonstrated, please provide verifiable evidence.)  **Community & Public Health Benefits**  *COMMUNITY & PUBLIC HEALTH BENEFITS - Health Activities & Products*  Community health services - Diagnostic, therapeutic and preventive health services provided for individuals in the community. Demonstrated/Potential (If demonstrated, please provide verifiable evidence.)  Consumer software - Digital and mobile technologies used by or for consumers to improve health care delivery and outcomes. Demonstrated/Potential (If demonstrated, please provide verifiable evidence.)  Health education resources - Educational resources that relate to the improvement of health for individuals, populations, or communities. Demonstrated/Potential (If demonstrated, please provide verifiable evidence.)  *COMMUNITY & PUBLIC HEALTH BENEFITS - Health Care Characteristics*  Health care accessibility - Equity and ability for all to gain entry to and to receive services from the health care system. Demonstrated/Potential (If demonstrated, please provide verifiable evidence.)  Health care delivery - Provision and distribution of health services to a patient population. Demonstrated/Potential (If demonstrated, please provide verifiable evidence.)  Health care quality - General characteristics of the health service or care provided based on accepted standards of quality. Demonstrated/Potential (If demonstrated, please provide verifiable evidence.)  *COMMUNITY & PUBLIC HEALTH BENEFITS - Health Promotion*  Disease prevention & reduction - Resources that enhance health promotion and disease prevention in communities or populations. Demonstrated/Potential (If demonstrated, please provide verifiable evidence.)  Life expectancy & quality of life - Life expectancy or quality of life for communities or populations. Demonstrated/Potential (If demonstrated, please provide verifiable evidence.)  Public health practices - Organization or delivery of public health services benefits to communities or populations. Demonstrated/Potential (If demonstrated, please provide verifiable evidence.)  **Economic Benefits**  *ECONOMIC BENEFITS - Commercial Products*  License agreements - Governmental permits based on intellectual property. Researchers develop new technologies to be used for diagnostic or therapeutic applications with possible commercial use. Examples of applications are vaccines, drug deliveries, medical devices, imaging, software, algorithms, blood and tissue products, cellular and gene therapy, among others. A license allows for exploration of applications for potential human benefit. Demonstrated/Potential (If demonstrated, please provide verifiable evidence.)  Non-profit or commercial entities - Creation of businesses or non-profit agencies. Demonstrated/Potential (If demonstrated, please provide verifiable evidence.)  Patents - Government authority or licenses based on intellectual property. Demonstrated/Potential (If demonstrated, please provide verifiable evidence.)  *ECONOMIC BENEFITS - Financial Savings & Benefits*  Cost effectiveness - Improvement in the benefits of a program relative to its cost. Demonstrated/Potential (If demonstrated, please provide verifiable evidence.)  Cost savings - Reduced financial costs of services or goods to providers or consumers. Demonstrated/Potential (If demonstrated, please provide verifiable evidence.)  Societal & financial cost of illness - Reduced social and economic costs of acute or chronic disease or other health conditions. Demonstrated/Potential (If demonstrated, please provide verifiable evidence.)  **Policy & Legislative Benefits**  *POLICY & LEGISLATIVE BENEFITS - Advisory Activities*  Committee participation - Participation in advisory, standards, or other governmental or nongovernmental committees. Demonstrated/Potential (If demonstrated, please provide verifiable evidence.)  Expert testimony - Presentation of data or results to governmental, judicial, or other regulatory bodies. Demonstrated/Potential (If demonstrated, please provide verifiable evidence.)  Scientific research reports - Research findings or summaries provided to inform policy or regulatory activity. Demonstrated/Potential (If demonstrated, please provide verifiable evidence.)  *POLICY & LEGISLATIVE BENEFITS - Policies & Legislation*  Legislation - Bills, laws, statutes, and ordinances passed through formal legislative bodies such as congress, state legislatures, and county and city councils. Demonstrated/Potential (If demonstrated, please provide verifiable evidence.)  Policies - Procedural rules formally adopted and mandated by governmental agencies or private or non-profit organizations. These include what are commonly referred to as big "P" policies (governmental) and little "p" policies (non-governmental organizational). Demonstrated/Potential (If demonstrated, please provide verifiable evidence.)  Standards - Formal designations of levels of quality defined by industry, occupational groups, or governmental bodies. Demonstrated/Potential (If demonstrated, please provide verifiable evidence.)  **Academic outcomes from your Pilot Award**  Did this Pilot grant result in peer-reviewed publications? Yes/No  Please list the full citations for the publications resulting from this CTSC Pilot Grant.  Did this Pilot grant result in subsequent extramural funding relating to the pilot project? Yes/No  Please list the extramural funding awards. Please include the funder, grant mechanism, dates,  title, and total amount funded.  **Other Benefits**  Are there other types of benefits from your CTSC Pilot research that are not mentioned above? If yes, please describe below.  (OPTIONAL) Upload a document to support your TS benefits.  Have you completed this pilot research? ("Completed" means you are no longer doing this research and no longer publishing or writing grants on this project)  No, research is still ongoing/Yes, research is completed |
| --- |
| **Translational Science Benefits from KL2 Research**  KL2 Research Title:  If the title or description of your KL2 research project (above, red text) is incorrect or needs updating, please write the correct title below. Otherwise, leave blank.  Are there other lines of research you were able to do during the protected time of the KL2 that were outside your main KL2 project? Please briefly list them here.  **In the sections below, please indicate any translational science benefits from any of the research you did during your KL2 training time. (It is NOT expected that you will have benefits in all domains)**  **Clinical & Medical Benefits**  *CLINICAL & MEDICAL BENEFITS - Procedures and Guidelines*  Diagnostic procedures - Methods and techniques performed to diagnose disease, disorders, or conditions.  Demonstrated/Potential (If demonstrated, please provide verifiable evidence.)  Investigative procedures - Research methods used in pre-clinical, clinical, and other scientific studies. Demonstrated/Potential (If demonstrated, please provide verifiable evidence.)  Guidelines - Formal recommendations or principles to assist with patient care for specific clinical circumstances. Demonstrated/Potential (If demonstrated, please provide verifiable evidence.)  Therapeutic procedures - Methods & techniques that pertain to interventions, treatment, or prevention of diseases, disorders or conditions. Demonstrated/Potential (If demonstrated, please provide verifiable evidence.)  *CLINICAL & MEDICAL BENEFITS - Tools & Products*  Biological factors & products - Biological substances used to indicate, diagnose, prevent, or treat diseases or medical conditions. Demonstrated/Potential (If demonstrated, please provide verifiable evidence.)  Biomedical technology - Technology applications for solving medical problems. Demonstrated/Potential (If demonstrated, please provide verifiable evidence.)  Drugs - Pharmaceutical products for human or veterinary use. Demonstrated/Potential (If demonstrated, please provide verifiable evidence.)  Equipment & supplies - Apparatus, instruments, and materials for diagnostic, surgical, therapeutic, and scientific procedures. Demonstrated/Potential (If demonstrated, please provide verifiable evidence.)  Software technologies - Computer programs or software installed on mobile or other electronic devices. Demonstrated/Potential (If demonstrated, please provide verifiable evidence.)  **Community & Public Health Benefits**  *COMMUNITY & PUBLIC HEALTH BENEFITS - Health Activities & Products*  Community health services - Diagnostic, therapeutic and preventive health services provided for individuals in the community. Demonstrated/Potential (If demonstrated, please provide verifiable evidence.)  Consumer software - Digital and mobile technologies used by or for consumers to improve health care delivery and outcomes. Demonstrated/Potential (If demonstrated, please provide verifiable evidence.)  Health education resources - Educational resources that relate to the improvement of health for individuals, populations, or communities. Demonstrated/Potential (If demonstrated, please provide verifiable evidence.)  *COMMUNITY & PUBLIC HEALTH BENEFITS - Health Care Characteristics*  Health care accessibility - Equity and ability for all to gain entry to and to receive services from the health care system. Demonstrated/Potential (If demonstrated, please provide verifiable evidence.)  Health care delivery - Provision and distribution of health services to a patient population. Demonstrated/Potential (If demonstrated, please provide verifiable evidence.)  Health care quality - General characteristics of the health service or care provided based on accepted standards of quality. Demonstrated/Potential (If demonstrated, please provide verifiable evidence.)  *COMMUNITY & PUBLIC HEALTH BENEFITS - Health Promotion*  Disease prevention & reduction - Resources that enhance health promotion and disease prevention in communities or populations. Demonstrated/Potential (If demonstrated, please provide verifiable evidence.)  Life expectancy & quality of life - Life expectancy or quality of life for communities or populations. Demonstrated/Potential (If demonstrated, please provide verifiable evidence.)  Public health practices - Organization or delivery of public health services benefits to communities or populations. Demonstrated/Potential (If demonstrated, please provide verifiable evidence.)  **Economic Benefits**  *ECONOMIC BENEFITS - Commercial Products*  License agreements - Governmental permits based on intellectual property. Researchers develop new technologies to be used for diagnostic or therapeutic applications with possible commercial use. Examples of applications are vaccines, drug deliveries, medical devices, imaging, software, algorithms, blood and tissue products, cellular and gene therapy, among others. A license allows for exploration of applications for potential human benefit. Demonstrated/Potential (If demonstrated, please provide verifiable evidence.)  Non-profit or commercial entities - Creation of businesses or non-profit agencies. Demonstrated/Potential (If demonstrated, please provide verifiable evidence.)  Patents - Government authority or licenses based on intellectual property. Demonstrated/Potential (If demonstrated, please provide verifiable evidence.)  *ECONOMIC BENEFITS - Financial Savings & Benefits*  Cost effectiveness - Improvement in the benefits of a program relative to its cost. Demonstrated/Potential (If demonstrated, please provide verifiable evidence.)  Cost savings - Reduced financial costs of services or goods to providers or consumers. Demonstrated/Potential (If demonstrated, please provide verifiable evidence.)  Societal & financial cost of illness - Reduced social and economic costs of acute or chronic disease or other health conditions. Demonstrated/Potential (If demonstrated, please provide verifiable evidence.)  **Policy & Legislative Benefits**  *POLICY & LEGISLATIVE BENEFITS - Advisory Activities*  Committee participation - Participation in advisory, standards, or other governmental or nongovernmental committees. Demonstrated/Potential (If demonstrated, please provide verifiable evidence.)  Expert testimony - Presentation of data or results to governmental, judicial, or other regulatory bodies. Demonstrated/Potential (If demonstrated, please provide verifiable evidence.)  Scientific research reports - Research findings or summaries provided to inform policy or regulatory activity. Demonstrated/Potential (If demonstrated, please provide verifiable evidence.)  *POLICY & LEGISLATIVE BENEFITS - Policies & Legislation*  Legislation - Bills, laws, statutes, and ordinances passed through formal legislative bodies such as congress, state legislatures, and county and city councils. Demonstrated/Potential (If demonstrated, please provide verifiable evidence.)  Policies - Procedural rules formally adopted and mandated by governmental agencies or private or non-profit organizations. These include what are commonly referred to as big "P" policies (governmental) and little "p" policies (non-governmental organizational). Demonstrated/Potential (If demonstrated, please provide verifiable evidence.)  Standards - Formal designations of levels of quality defined by industry, occupational groups, or governmental bodies. Demonstrated/Potential (If demonstrated, please provide verifiable evidence.)  Would you be willing to be interviewed via Zoom about your translational science benefits?  (This is to better assess how your work might be written up as a Translational Science Case Study - with you as one of the authors.) Yes/No  **Other Benefits**  During your KL2 training, have you trained graduate students, post-doctoral students, research associates, or other personnel in Clinical and Translational Science? If yes, please list their names and the positions they held while training with you.  (*NOTE: Please mark them with an * if they are from a group that is underrepresented in research. The following racial and ethnic groups have been shown to be underrepresented in biomedical research: Blacks or African Americans, Hispanics or Latinos, American Indians or Alaska Natives, Native Hawaiians, and other Pacific Islanders. NIH 2022)  (Hint: you can copy/paste from your CV training section.)  Is there another type of benefit from your research that is not mentioned above? If yes, please describe below.  Are you still working in the same general field of research as you were during your KL2 project? Yes/No  What is your new field of research? |

## Appendix B: Interview Protocol

| **Interview Question** | **Purpose** |
| --- | --- |
| 1. How did the investigators use the CTSC? | To understand the engagement of researchers with the CTSC and its role in supporting their work. This includes how access to resources like funding, mentorship, infrastructure, or collaborative networks facilitated progress and enhanced the societal relevance of their research. |
| 2. In which TSBM categories or indicators did the research demonstrate societal benefits? | To identify the broader societal impacts of the research using the Translational Science Benefits Model (TSBM) framework. This includes contributions to improved healthcare delivery, public health outcomes, policy changes, and other measurable benefits. |
| 3. What populations were affected by the research, and where geographically? | To explore the demographic and geographic scope of the research’s impact, identifying who was affected and where. |
| 4. How did the research improve public health? | To evaluate the research’s contributions to public health improvements, such as increased access to care and better health outcomes, especially for high-risk communities. |
| 5. Is the research being expanded to additional populations? If so, how and which populations? | To determine whether the research is being extended to benefit new groups. This includes understanding expansion strategies, targeted populations, and the potential societal impacts. |
| 6. What challenges and facilitators were encountered during the research? | To identify obstacles (e.g., resource limitations, systemic issues, technical barriers) and factors that facilitated success (e.g., support, partnerships, innovative approaches). |
| 7. What are the next steps for this research? | To outline planned future directions, such as scaling efforts, involving new stakeholders, or initiating new research questions, with an emphasis on amplifying impact and broadening implications. |
